# High Trait Self-Control and Low Boredom Proneness Help COVID-19 Homeschoolers

**DOI:** 10.3389/fpsyg.2021.594256

**Published:** 2021-02-18

**Authors:** Corinna S. Martarelli, Simona G. Pacozzi, Maik Bieleke, Wanja Wolff

**Affiliations:** ^1^Faculty of Psychology, Swiss Distance University Institute, Brig, Switzerland; ^2^Department for Psychology of Development and Education, Faculty of Psychology, University of Vienna, Vienna, Austria; ^3^Department of Sport Science, University of Konstanz, Konstanz, Germany; ^4^Department of Educational Psychology, University of Bern, Bern, Switzerland

**Keywords:** COVID-19, homeschooling, boredom, self-control, self-regulation

## Abstract

In response to the coronavirus disease 2019 (COVID-19) schools around the world have been closed to protect against the spread of coronavirus. In several countries, homeschooling has been introduced to replace classroom schooling. With a focus on individual differences, the present study examined 138 schoolers (age range = 6 to 21 years) regarding their self-control and boredom proneness. The results showed that both traits were important in predicting adherence to homeschooling. Schoolers with higher levels of self-control perceived homeschooling as less difficult, which in turn increased homeschooling adherence. In contrast, schoolers with higher levels of boredom proneness perceived homeschooling as more difficult, which in turn reduced homeschooling adherence. These results partially hold when it comes to studying in the classroom. However, boredom threatened adherence only in the homeschooling context. Our results indicate that boredom proneness is a critical construct to consider when educational systems switch to homeschooling during a pandemic.

## Introduction

“*And the faculty of voluntarily bringing back a wandering attention, over and over again, is the very root of judgment, character, and will. No one is compos sui [master of himself] if he have it not. An education which should improve this faculty would be the education per excellence.” William James, The Principles of Psychology (1980)*.

The coronavirus disease 2019 (COVID-19) pandemic represents a global crisis affecting nearly all countries and every sector of human life including health, economy, and education. Current theorizing highlights the relevance of social and behavioral sciences in order to contain the spread of the virus (Van Bavel et al., 2020). To slow the spread of COVID-19, countries have employed various social distancing (or physical distancing) measures. One frequently employed social distancing measure is the closing of schools and educational institutions. In March 2020, such measures were employed by 102 countries, with the effect of almost 900 million children and adolescents being asked to switch to homeschooling (Burn, 2020). This forced COVID-19 homeschooling should not be mistaken for classical homeschooling, which has emerged since the late 1970s as a dedicated educational approach (Jolly and Matthews, 2020). Contrary to classical homeschooling, the current situation was imposed to schoolers, parents, and teachers, and there was a great variation in terms of restrictions and in terms of teaching activities in general (e.g., online teaching, homeschooling, self-organized study; Thorell et al., 2020). As it is likely that homeschooling presents schoolers with challenges that differ from those that they experience during classroom schooling, it is crucial to investigate the role of individual differences in explaining children's struggles and successes in dealing with this change in educational format. Specifically, given the reduction in external structure, we expect homeschooling to present a self-regulatory challenge. In turn, we expect personality traits that affect how people deal with such challenges to be of particular importance during this time. Boredom proneness and self-control are two such traits. Here, we draw upon recent functional theorizing on boredom and self-control (Wolff and Martarelli, 2020) to investigate the role of boredom proneness and trait self-control in how schoolers cope with the challenges of homeschooling in the current situation. Our approach can help in identifying schoolers that might struggle with homeschooling and enable the development of strategies that enhance homeschooling adherence.

### Self-Control and Boredom

Self-control and boredom are closely linked: High self-control has shown to be associated with low boredom proneness (Isacescu et al., 2017; Mugon et al., 2018; Bieleke et al., 2020b; Boylan et al., 2020; Wolff et al., 2020b). In addition, boredom seems to be related with poor cognitive, physical and emotional self-regulation (Isacescu et al., 2017; Wolff et al., 2020a) and while high trait self-control is rather consistently linked with positive outcomes (Moffitt et al., 2011), boredom proneness is linked with mostly negative outcomes (Eastwood et al., 2012). Based on this inverse relationship on the trait level, recent theoretical work suggests that the sensation of boredom and the sensation that accompanies the application of self-control (i.e., effort) both act as functional signals that help people orient their behavior (Wolff and Martarelli, 2020), which has been supported by empirical research (Bieleke et al., 2020a). According to this perspective, the perception of effort that arises when self-control is applied signals the costs of a current activity (e.g., the cost of foregoing the gratification of surfing the internet and to do the math assignment instead) and if the cost outweighs the activity's benefit, then this activity is terminated (Kurzban et al., 2013). Within this line of reasoning, boredom is understood as a signal that the task at hand is not worth pursuing (e.g., completing the math assignment) and that there might be other more rewarding activities (e.g., surfing the internet). Thus, boredom is a strong driver of behavioral change (Geana et al., 2016; Gomez-Ramirez and Costa, 2017; Bench and Lench, 2019; Danckert, 2019). To resist the boredom-induced drive for change, self-control is required (Milyavskaya et al., 2019), thereby making boredom an additional self-control demand, that schoolers are likely to have to deal with during homeschooling.

Self-control can be described as the effort individuals exert to overcome predominant responses to reach a goal (Shenhav et al., 2013) and has been found to emerge early in life and to continue to develop until adulthood (Pan and Zhu, 2018). Self-control is required in many situations and higher levels of self-control have been shown to be associated with several positive outcomes including transition to school and academic achievement (Tangney et al., 2004; Welsh et al., 2010; Zhu et al., 2016; Lindner et al., 2018; Martarelli et al., 2018). Given the key role of self-control in the educational context, it is plausible that high self-control helps schooler to better deal with specific demands of homeschooling (Blume et al., 2020). Indeed, studying in a more unstructured context such as at home where other (more rewarding) activities are within the reach of schoolers, possibly requires more effortful control than studying in a classroom. Thus, the role of self-control might be even more pronounced during homeschooling than in the classroom setting. In turn, to keep on studying while in home confinement might be particularly challenging for schoolers with low levels of self-control.

Boredom has been defined as the inability to successfully engage attention with the outer world or inner worlds (Eastwood et al., 2012) and has been associated with several negative outcomes also in the academic context (Bearden et al., 1989; Pekrun et al., 2010). While the detrimental effects of boredom in the classroom might partially be relieved by the teacher (e.g., by changing the task at hand, by redirecting attention to learning requirements, and more generally by highlighting the *meaning* of studying, or by adapting the *difficulty* of the learning material; Westgate and Wilson, 2018), homeschoolers might be challenged by several other opportunities (e.g., surfing the internet) and the experience of boredom will enhance the saliency of these other more rewarding activities (Elhai et al., 2017). Moreover, being in an environment that offers many alternative activities and not being supposed to engage with these activities is perceived as particularly boring (Struk et al., 2020). Thus, we predict that boredom prone schoolers will find it more difficult to study in homeschooling and some will mitigate boredom by engaging in alternative activities.

### The Present Study

**Figure 1:**
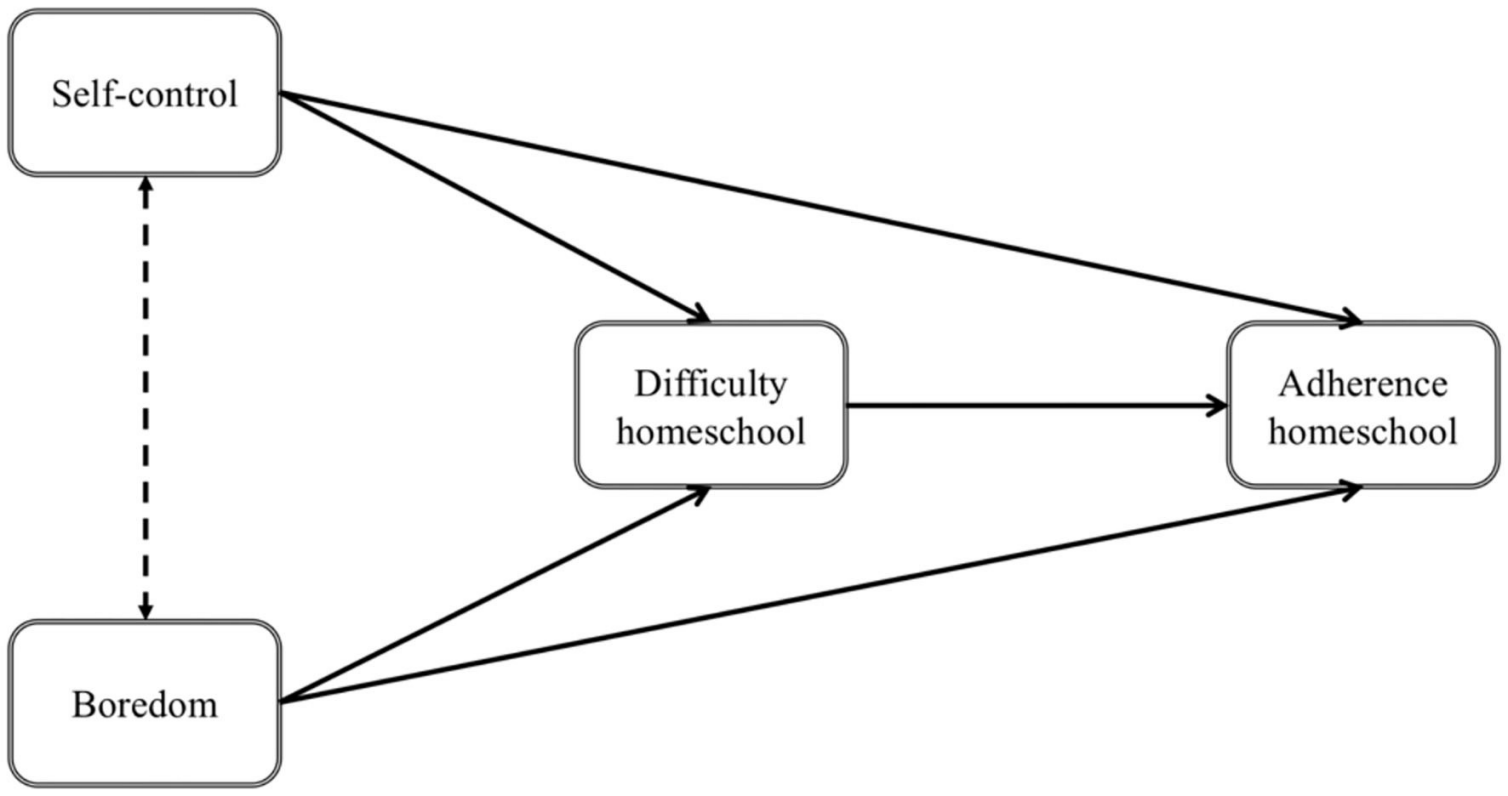
Conceptual model in which the effects of self-control and boredom on adherence are mediated by the perceived difficulty of homeschooling.

The rapid and exponential spread of the coronavirus across the world has generated substantial economic and social disruptions that have affected people's lives and well-being. For schoolers, parents and teachers school closures and the associated homeschooling has been a new challenge. The schoolers' perspective quickly gets out of focus when discussing about homeschooling. Therefore, we developed a short, child-friendly questionnaire, which should give information about the experiences of schoolers. Based on recent theorizing about self-control and boredom (Martarelli and Wolff, 2020; Wolff and Martarelli, 2020) we aimed to investigate the role of trait self-control and boredom proneness in the perceived difficulty of homeschooling and in the adherence to homeschooling. We expect that schoolers high in self-control will find it easier to study in homeschooling, which in turn should increase homeschooling adherence. Further, we expect that schoolers high in trait boredom proneness find it more difficult to study in homeschooling, which in turn should decrease homeschooling adherence. We expect trait self-control and boredom proneness to have direct effects on homeschooling adherence. Finally, we expect similar relationships between these variables in the classroom; however, because of how classroom lectures are structured these relationships should be less pronounced. Figure 1 summarizes the proposed mediation model.

## Materials and Methods

### Participants

One hundred thirty-eight schoolers (58.7% female) took part to the study. All of them were attending school (44.9% primary school, 19.6% secondary school, 32.6% college, 2.9% other) and in homeschooling when filling in the questionnaire (end of April 2020). The age range was between 6 and 21 years and the vast majority of the participating schoolers lived in Switzerland (98.6%). More than half of the participants were French-speaking (55.1%), the remaining participants were German-speaking. About half of the sample reported their parents held a university degree (49.28%). The schoolers who participated in this study were contacted via their parents, who first gave parental consent. The local ethics committee approved the study, which was conducted in accordance with the Declaration of Helsinki.

### Procedure

Schoolers completed the questionnaire online, using the freely available open source software Limesurvey (www.limesurvey.org). All schoolers gave written informed consent, and they could ask parents for help, if needed. The study lasted on average 10 min and we used items with 5-point Likert scales throughout the study (1 = *do not agree at all*, 5 = *fully agree*) with the exception of demographics.

First, *adherence to study in homeschooling* was assessed with one item (“At home, I reliably work on the tasks that the teacher gives me”) and *perceived difficulty of homeschooling* was measured with a set of three items (“I find it hard to study in homeschooling,” “I find it boring to study in homeschooling,” “I have no desire to study in homeschooling”). Next, the same questions were asked for the classroom schooling context before the COVID-19 pandemic. We assessed *adherence to study in the classroom* (“In the classroom I reliably work on the tasks that the teacher gives me”) and *perceived difficulty of studying in the classroom* with a set of three items (“I find it hard to study in the classroom,” “I find it boring to study in the classroom,” “I have no desire to study in the classroom”).

Subsequently, schoolers worked on five items taken from the German short version of the *Self-Control* Scale (Tangney et al., 2004; Bertrams and Dickhäuser, 2009), which measures individuals' general ability to regulate themselves (e.g., “Sometimes I can't stop myself from doing something, even if I know it is wrong”), and four questions taken from the German Short *Boredom Proneness* Scale (Struk et al., 2017; Martarelli et al., 2020), which measures individuals' boredom proneness (e.g., “I find it hard, to entertain myself”). The wording of items was adapted to children and is reported in the Supplementary Material. Finally, schoolers reported demographics, including gender, age, residence, attended school, and parents' educational level.

## Results

To test the proposed model (Figure 1) we used *R* (R core Team, 2020) with the functionalities of the *lavaan* package (Rosseel et al., 2018). The standard errors for the defined parameters were computed with bias-corrected bootstrap method (1,000 bootstrap repetitions). The R code to reproduce analyses as well as the data set is available on OSF at https://osf.io/32rwe/. Descriptive statistics were computed with jamovi version 1.1.9.0 (The jamovi project, 2020) and with JASP version 0.12.2 (JASP Team, 2020).

### Descriptive Statistics

Adherence to study in homeschooling during the COVID-19 situation was relatively high (mean of 3.84 out of 5) but significantly lower than adherence to study in the classroom (mean of 4.36 out of 5), paired-samples *t-*test *t*_(137)_ = 5.70, *p* < .001, Cohen's *dz* =0.48. The internal consistency of the scales was good, skewness and kurtosis were low (see Table 1).

**Table 1:** Descriptive summary of measures.

**Measure**	**Mean (SD)**	**Skewness**	**Kurtosis**	**Alpha**	**Omega**
Self-control	3.23 (0.81)	0.13	−0.82	0.709	0.710
Boredom	2.13 (0.87)	0.77	0.29	0.790	0.796
Difficulty homeschool	2.95 (1.07)	−0.15	−0.82	0.773	0.776
Difficulty classroom	2.32 (1.03)	0.56	−0.51	0.850	0.851
Adherence homeschool	3.84 (1.19)	−0.94	0.07		
Adherence classroom	4.36 (0.76)	−1.32	2.40		

*N = 138*.

With regard to preliminary correlation analyses, Table 2 shows significant correlations between our predictor, mediator and outcome variables. The correlations were in the expected direction, and stronger in the homeschooling context when compared to the classroom.

**Table 2:** Correlations of measures.

		**Self-control**	**Boredom**	**Difficulty**	**Adherence**
Self-control	Pearson's *r*	–	−0.413	−0.414	0.360
	*p*-value	–	<0.001	<0.001	<0.001
Boredom	Pearson's *r*	−0.413	–	0.315	−0.238
	*p*-value	<0.001	–	<0.001	0.005
Difficulty	Pearson's *r*	−0.487	0.342	–	−0.270
	*p*-value	<0.001	<0.001	–	0.001
Adherence	Pearson's *r*	0.595	−0.395	−0.483	–
	*p*-value	<0.001	<0.001	<0.001	–

*N = 138. Correlations and significance for the homeschooling model are reported in the lower part of the table and correlations for the classroom model are reported in the upper part of the table*.

### Homeschooling Model

**Figure 2:**
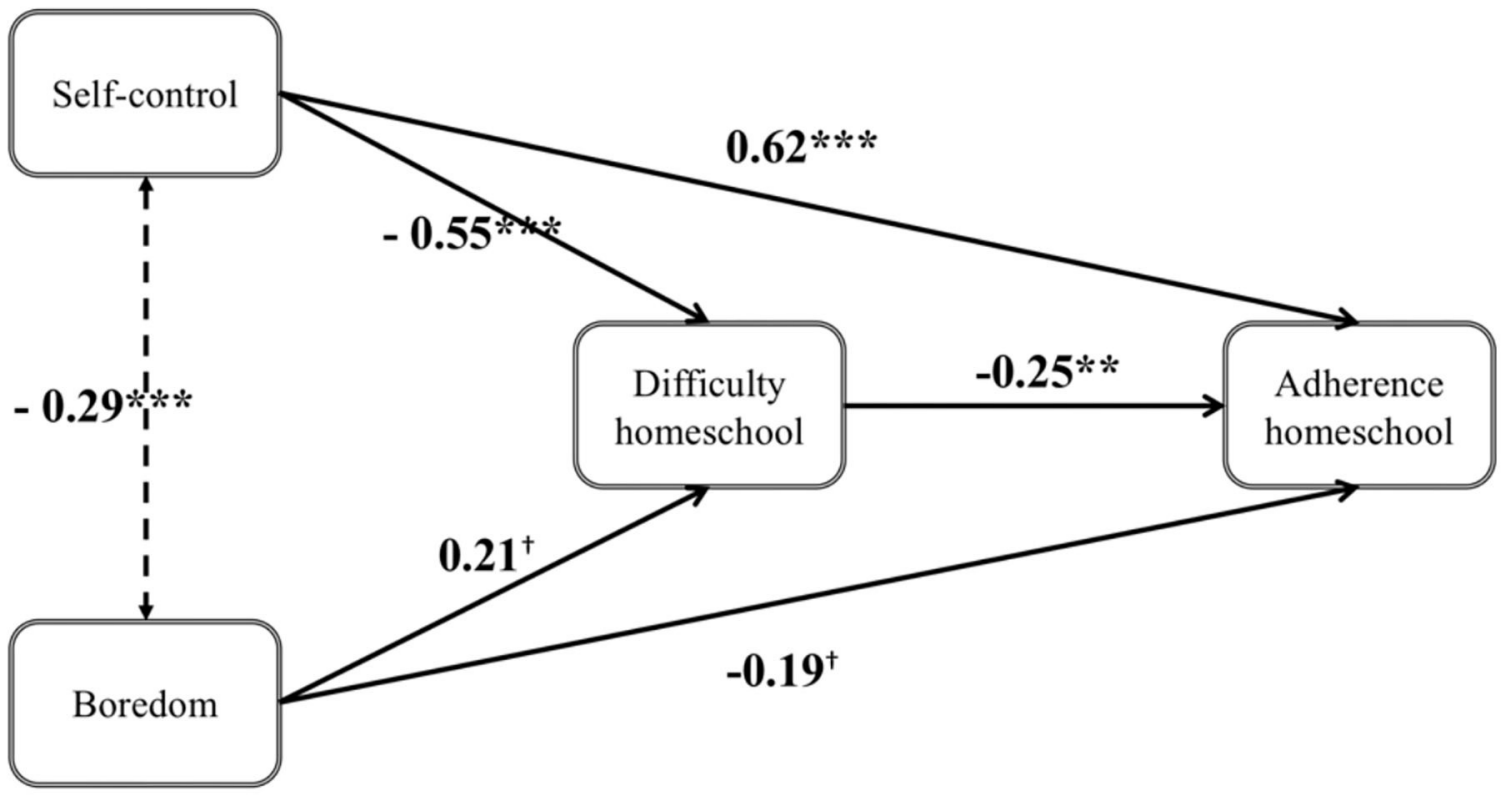
Homeschooling model *N* = 138. Coefficients are unstandardized ^***^. Difference is significant at the 0.001 level (two-tailed) and ^**^. Difference is significant at the 0.01 level (two-tailed) ^†^. Difference is significant at the 0.1 level (two-tailed).

The unstandardized and standardized path coefficients, standard errors, and *p*-values of the homeschooling model can be found in Table 3 and are depicted in Figure 2. Individual differences in self-control and boredom influenced the perceived difficulty of homeschooling, self-control: *b* = −0.550, β = −0.417, *SE* = 0.106, *p* = < 0.001; boredom: *b* = 0.208, β = 0.170, *SE* = 0.109, *p* = 0.056. Both variables together explained 26.2% of variance in perceived difficulty. The difficulty of homeschooling in turn negatively influenced the adherence to homeschooling, *b* = −0.252, β = −0.227, *SE* = 0.078, *p* = 0.001. Individual differences in self-control and boredom also influenced the adherence to homeschooling, self-control: *b* = 0.624, β = 0.426, *SE* = 0.114, *p* = < 0.001; boredom: *b* = −0.192, β = −0.142, *SE* = 0.111, *p* = 0.083. For self-control not only the direct effect, but also the indirect effect, *b* = 0.139, β = 0.095, *SE* = 0.052, *p* = 0.007, and the total effect, *b* = 0.762, β = 0.521, *SE* = 0.110, *p* = < 0.001, were significant. The indirect effect of boredom on adherence was not significant, *b* = −0.052, β = −0.039, *SE* = 0.033, *p* = 0.114, while the total effect turned out to be significant, *b* = −0.245, β = −0.180, *SE* = 0.114, *p* = 0.031. The model explained 42.0% of variance in adherence to homeschooling.

**Table 3:** Homeschooling Model.

**Type**	**Effect**	**Estimate**	**Std. Err**	**95% C.I. lower**	**95% C.I. upper**	***z*-value**	***p*-value**	**Std. estimate**
Component	Difficulty ⇒ Adherence	−0.252	0.078	−0.406	−0.096	−3.241	0.001	−0.227
Direct	Self-control ⇒ Adherence	0.624	0.114	0.387	0.842	5.470	<0.001	0.426
Direct	Boredom ⇒ Adherence	−0.192	0.111	−0.415	0.014	−1.734	0.083	−0.142
Component	Self-control ⇒ Difficulty	−0.550	0.106	−0.755	−0.340	−5.200	<0.001	−0.417
Component	Boredom ⇒ Difficulty	0.208	0.109	0.003	0.442	1.910	0.056	0.170
Covariance	Boredom ~~ self-control	−0.290	0.058	−0.415	−0.181	−5.014	<0.001	−0.413
Variance	Adherence	0.810	0.109	0.634	1.077	7.398	<0.001	0.580
Variance	Difficulty	0.836	0.087	0.679	1.023	9.655	<0.001	0.738
Variance	Self-control	0.652	0.061	0.546	0.787	10.736	<0.001	1.000
Variance	Boredom	0.758	0.098	0.579	0.979	7.752	<0.001	1.000
Indirect	Self-control ⇒ Difficulty ⇒ Adherence	0.139	0.052	0.056	0.263	2.686	0.007	0.095
Indirect	Boredom ⇒ Difficulty ⇒ Adherence	−0.052	0.033	−0.145	−0.005	−1.581	0.114	−0.039
Total	Self-control ⇒ Adherence	0.762	0.110	0.536	0.972	6.935	<0.001	0.521
Total	Boredom ⇒ Adherence	−0.245	0.114	−0.471	−0.027	−2.153	0.031	−0.180

*N = 138. Standard errors were computed with bias-corrected bootstrap method (1,000 bootstrap repetitions)*.

We additionally estimated the model including age and gender. Gender (coded as 0 for male and 1 for female) had a significant impact on self-control, *b* = 0.290, β = 0.177, *SE* = 0.124, *p* = 0.020. Age had a significant impact on self-control, *b* = −0.075, β = −0.373, *SE* = 0.017, *p* < 0.001, on boredom, *b* = 0.070, β = 0.319, *SE* = 0.018, *p* < 0.001, and on adherence to homeschooling, *b* = −0.041, β = −0.139, *SE* = 0.021, *p* = 0.049. Gender and age did not change the other results (no change in accepting or rejecting the null hypotheses).

### Classroom Model

**Figure 3:**
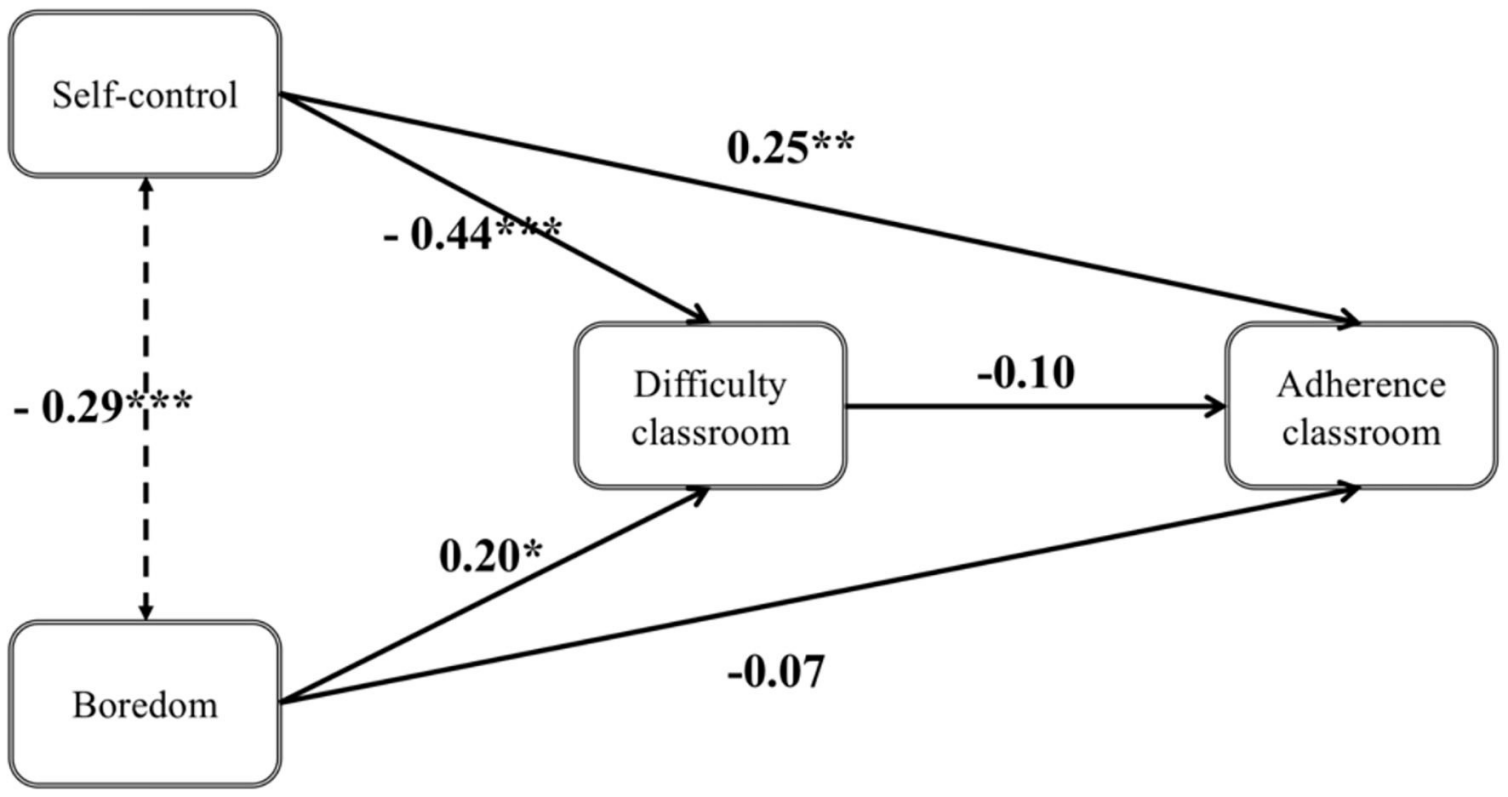
Classroom model *N* = 138. Coefficients are unstandardized ^***^. Difference is significant at the 0.001 level (two-tailed) and ^**^. Difference is significant at the 0.01 level (two-tailed).

The unstandardized and standardized path coefficients, standard errors and *p*-values of the classroom model can be found in Table 4 and are depicted in Figure 3. Individual differences in self-control and boredom influenced the perceived difficulty of studying in the classroom, self-control: *b* = −0.436, β = −0.342, *SE* = 0.100, *p* = < 0.001; boredom: *b* = 0.205, β = 0.174, *SE* = 0.101, *p* = 0.043. Both variables together explained 19.6% of variance in perceived difficulty. However, the difficulty of studying in the classroom did not influence the adherence to studying in the classroom, *b* = −0.098, β = −0.132, *SE* = 0.071, *p* = 0.167. The direct effect of self-control on adherence to studying in the classroom, *b* = 0.254, β = 0.270, *SE* = 0.093, *p* = 0.006 turned out to be significant, while the indirect effect was not significant, *b* = −0.020, β = −0.023, *SE* = 0.019, *p* = 0.284. The total effect of self-control on adherence was significant, *b* = 0.297, β = 315, *SE* = 0.081, *p* = < 0.001. The direct, indirect and total effect of boredom on adherence to studying in the classroom were not significant (*ps* > 0.284). The model explained 15.3% of variance in adherence to studying in the classroom.

**Table 4:** Classroom model.

**Type**	**Effect**	**Estimate**	**Std. Err**	**95% C.I. lower**	**95% C.I. upper**	**z-value**	**p-value**	**Std. estimate**
Component	Difficulty ⇒ Adherence	−0.098	0.071	−0.240	0.046	−1.381	0.167	−0.132
Direct	Self-control ⇒ Adherence	0.254	0.093	0.064	0.427	2.731	0.006	0.270
Direct	Boredom ⇒ Adherence	−0.074	0.096	−0.256	0.118	−0.776	0.438	−0.085
Component	Self-control ⇒ Difficulty	0.436	0.100	−0.628	−0.243	−4.357	<0.001	−0.342
Component	Boredom ⇒ Difficulty	0.205	0.101	0.004	0.406	2.027	0.043	0.174
Covariance	Boredom ~~ self-control	−0.290	0.058	−0.415	−0.181	−5.014	<0.001	−0.413
Variance	Adherence	0.490	0.091	0.352	0.726	5.378	<0.001	0.847
Variance	Difficulty	0.848	0.092	0.697	1.082	9.173	<0.001	0.804
Variance	Self-control	0.652	0.061	0.546	0.787	10.736	<0.001	1.000
Variance	Boredom	0.758	0.098	0.579	0.979	7.752	<0.001	1.000
Indirect	Self-control ⇒ Difficulty ⇒ Adherence	0.042	0.035	−0.014	0.124	1.225	0.221	0.045
Indirect	Boredom ⇒ Difficulty ⇒ Adherence	−0.020	0.019	−0.076	0.005	−1.072	0.284	−0.023
Total	Self-control ⇒ Adherence	0.297	0.081	0.131	0.445	3.669	<0.001	0.315
Total	Boredom ⇒ Adherence	−0.094	0.097	−0.278	0.103	−0.976	0.329	−0.108

*N = 138. Standard errors were computed with bias-corrected bootstrap method (1,000 bootstrap repetitions)*.

We additionally estimated the model including age and gender. Gender (coded as 0 for male and 1 for female) had a significant impact on self-control, *b* = 0.290, β = 0.177, *SE* = 0.124, *p* = 0.020. Age had a significant impact on self-control, *b* = −0.075, β = −0.373, *SE* = 0.017, *p* < 0.001, on boredom, *b* = 0.070, β = 0.319, *SE* = 0.018, *p* < 0.001, and on adherence to studying in the classroom, *b* = −0.038, β = −0.198, *SE* = 0.019, *p* = 0.042. When adjusting for age and gender, we found no changes in the pattern of results with one exception: the direct effect of self-control just missed significance, *b* = 0.198, β = 0.210, *SE* = 0.105, *p* = 0.060.

## Discussion

Here, we show that both trait self-control and boredom proneness predict adherence to homeschooling (according to the significance of their total effects and the model's explained variance). Schoolers with higher levels of self-control perceived homeschooling as less difficult, which in turn increased homeschooling adherence. In contrast, schoolers with higher levels of boredom proneness found it more difficult to study in homeschooling (effect is significant at the 0.1 level) and showed less adherence to homeschooling (effect is significant at the 0.1 level). The same pattern of association partially occurred in the classroom context. Schoolers with higher levels of self-control perceived studying in the classroom as less difficult and showed an increase in adherence. However, boredom proneness did not threaten adherence in the classroom (direct, indirect and total effects of boredom turned out to be non-significant in the classroom model). Moreover, there was a large difference in explained variance (42.0% in the homeschooling model vs. 15.3% in the classroom model).

According to these results, schoolers with low levels of self-control and high levels of boredom proneness might be disadvantaged in a homeschooling setting. Generally these findings fit well with recent theoretical work ascribing a key role to self-control and boredom as guiding signals of goal-directed behavior (Wolff and Martarelli, 2020) and with recent empirical work showing a strong link between self-control and boredom (e.g., Isacescu et al., 2017). Thus, schoolers, who tend to get bored also tend to show low self-control and this structural combination could make the continuation of their education during a pandemic challenging.

In order to create equal educational opportunities, teachers, parents, and school principals should take boredom into account in the context of homeschooling during a pandemic. One possibility to reduce the detrimental effects of boredom on learning could be to create an environment conducive to learning. First, (more rewarding) behavioral alternatives that are not linked with studying should be removed. This suggestion builds upon a recent study of Struk et al. (2020) that investigated the impact of behavioral restrictions on boredom. The results showed that a room with no behavioral alternatives (when compared with a room containing several behavioral possibilities such as a computer with an open browser) was perceived as less boring by participants that were asked to entertain themselves with their thoughts. Moreover, participants in the room with behavioral alternatives more often engaged in these alternatives even though they were explicitly told not to do so. In other words, to mitigate boredom, they changed behavior (Struk et al., 2020). This behavioral pattern might also possibly occur in a homeschooling context. Thus, a learning environment that limits boredom could be achieved by proposing structured and diverse learning material, by using different learning modalities, and by reducing general distractions (e.g., putting distractions such as smartphones out of reach). Further, the difficulty of the learning material should be considered (Eastwood et al., 2012; Westgate and Wilson, 2018). By providing sufficient assistance, adapting the requirements or encouraging schoolers to seek for help, an under/overload can be prevented which in turn decreases the risk for a lack of adherence. Finally, boredom also arises as a consequence of lack of meaning (Van Tilburg and Igou, 2017; Westgate and Wilson, 2018). Thus, a further strategy might be to make homeschooling more meaningful and valuable.

Importantly, even in optimal environments (e.g., structured environment) people can still get bored. This is particularly true for boredom prone schoolers, who tend to experience boredom more frequently. Therefore, it would be crucial to equip schoolers with specific skills to recognize and cope with their boredom. One suggestion is, for e.g., to help schoolers to identify their individual “warning signs” of boredom (and of related constructs such as mind-wandering) and to encourage them to change activity by explicitly choosing the next activity, as for example to explore alternative learning material when bored with the task at hand. More generally, learning to recognize and use boredom in an adaptive manner might be useful for schoolers to redirect their attention to relevant external and/or internal information. This strategy should in turn also make homeschooling less self-control demanding.

There are limitations of the study that should be considered when interpreting the findings. One major limitation is that we used cross-sectional data, thus the temporal ordering of variables in the causal chain of mediation reflects theoretical considerations that do not necessarily correspond to how these effects unfold over time. For this reason, the estimated mediation effect is of correlational nature. Since correlations cannot offer insight into directionality of a relation between variables, it will be important for future research to carefully examine the directionality of the effect. Only then effective interventions can be implemented to improve homeschooling conditions. Furthermore, we worked with a small set of self-report measures, the sample size is also relatively small, and we recruited a convenience sample. Further research is needed to investigate other variables that are known to correlate with the variables measured here, such as self-efficacy or sense of agency (in terms of individual awareness of what one can do). Self-efficacy pertains to subjective expectations of being able to deal with situations that are novel and/or challenging on the basis of one's own competencies (Bandura, 1997). It is thus conceivable that self-efficacy would allow shedding additional light on the link between the perceived difficulty of homeschooling and adherence to homeschooling. Future research would benefit from investigating the generalizability in more detail using other methodologies (such as online experience sampling methods) and other samples, including different socio-cultural contexts.

Because boredom has been associated with numerous negative outcomes (Eastwood et al., 2012), it has a rather negative connotation. However, it has also been linked to positive outcomes such as creativity (Harris, 2000) and pro-social behavior (Van Tilburg and Igou, 2017). Boredom is a powerful motivator for negative and positive behaviors alike (Elpidorou, 2014, 2017). If correctly taken into account, boredom informs schoolers, that they are failing to pay attention and that a change in behavior is needed. One advantage of homeschooling (compared to traditional settings) might be the possible individualization of learning. Homeschoolers are more flexible and can thus more strongly regulate their way of learning if provided with the right support.

## Data Availability Statement

All relevant data are available on the Open Science Framework (https://osf.io/32rwe/).

## Ethics Statement

The study was reviewed and approved by the ethics commission of the Swiss Distance University Institute. Written informed consent from the participants' legal guardian was not required to participate in this online study in accordance with the institutional requirements.

## Author Contributions

CM, SP, MB, and WW contributed to conception and design of the study. SP and CM prepared the study and collected the data. CM performed the analyses and wrote the first draft of the manuscript. All authors discussed the results, contributed to the final manuscript and approved the submitted version.

## Conflict of Interest

The authors declare that the research was conducted in the absence of any commercial or financial relationships that could be construed as a potential conflict of interest.
