# Supplementary material for: High Trait Self-Control and Low Boredom Proneness Help COVID-19 Homeschoolers
Source: Front Psychol. 2021 Feb 18;12:594256. doi: 10.3389/fpsyg.2021.594256 (PMC7930236; doi:10.3389/fpsyg.2021.594256)
Supplement: Supplementary file 1 [file Table_1.docx]

Supplementary Material

# Supplementary Material

*Adapted items used to measure self-control trait and boredom proneness*

| **Self-control Trait** |
| --- |
| *SCS3r - Ich bin faul.* |
| *SCS5r - Ich tue manchmal Dinge, die schlecht für mich sind, wenn sie mir Spass machen.* |
| *SCS7r - Angenehme Aktivitäten und Vergnügen hindern mich manchmal daran, meine Arbeit zu machen.* |
| *SAS8r - Es fällt mir schwer, mich zu konzentrieren.* |
| *SCS10r - Manchmal kann ich mich selbst nicht daran hindern, etwas zu tun, obwohl ich weiss, dass es falsch ist.* |
| **Boredom Proneness** |
| *SBPS2 - Es fällt mir schwer, mich selbst zu unterhalten.* |
| *SBPS5 - Die meisten Dinge, die ich mache, motivieren mich nicht.* |
| *SBPS6 - In den meisten Situationen fällt es mir schwer, etwas zu finden, was ich tun oder sehen kann, um mein Interesse aufrechtzuerhalten.* |
| *SBPS7 - Einen Grossteil der Zeit sitze ich einfach rum und mache nichts.* |
